# A comprehensive network and pathway analysis of candidate genes in major depressive disorder

**DOI:** 10.1186/1752-0509-5-S3-S12

**Published:** 2011-12-23

**Authors:** Peilin Jia, Chung-Feng Kao, Po-Hsiu Kuo, Zhongming Zhao

**Affiliations:** 1 Department of Biomedical Informatics, Vanderbilt University School of Medicine, Nashville, TN, USA; 2 Department of Psychiatry, Vanderbilt University School of Medicine, Nashville, TN, USA; 3 Department of Public Health & Institute of Epidemiology and Preventive Medicine, College of Public Health, National Taiwan University, Taipei, Taiwan; 4 Research Center for Genes, Environment and Human Health, National Taiwan University, Taipei, Taiwan; 5 Department of Cancer Biology, Vanderbilt University School of Medicine, Nashville, TN, USA

## Abstract

**Background:**

Numerous genetic and genomic datasets related to complex diseases have been made available during the last decade. It is now a great challenge to assess such heterogeneous datasets to prioritize disease genes and perform follow up functional analysis and validation. Among complex disease studies, psychiatric disorders such as major depressive disorder (MDD) are especially in need of robust integrative analysis because these diseases are more complex than others, with weak genetic factors at various levels, including genetic markers, transcription (gene expression), epigenetics (methylation), protein, pathways and networks.

**Results:**

In this study, we proposed a comprehensive analysis framework at the systems level and demonstrated it in MDD using a set of candidate genes that have recently been prioritized based on multiple lines of evidence including association, linkage, gene expression (both human and animal studies), regulatory pathway, and literature search. In the network analysis, we explored the topological characteristics of these genes in the context of the human interactome and compared them with two other complex diseases. The network topological features indicated that MDD is similar to schizophrenia compared to cancer. In the functional analysis, we performed the gene set enrichment analysis for both Gene Ontology categories and canonical pathways. Moreover, we proposed a unique pathway crosstalk approach to examine the dynamic interactions among biological pathways. Our pathway enrichment and crosstalk analyses revealed two unique pathway interaction modules that were significantly enriched with MDD genes. These two modules are neuro-transmission and immune system related, supporting the neuropathology hypothesis of MDD. Finally, we constructed a MDD-specific subnetwork, which recruited novel candidate genes with association signals from a major MDD GWAS dataset.

**Conclusions:**

This study is the first systematic network and pathway analysis of candidate genes in MDD, providing abundant important information about gene interaction and regulation in a major psychiatric disease. The results suggest potential functional components underlying the molecular mechanisms of MDD and, thus, facilitate generation of novel hypotheses in this disease. The systems biology based strategy in this study can be applied to many other complex diseases.

## Background

During the past decade, rapid advances in high throughput technologies have helped investigators generate numerous genetic and genomic datasets, aiming to uncover disease causal genes and their actions in complex diseases. These datasets are often heterogeneous and multi-dimensional; thus, it is difficult to find consistent genetic signals for the connection to the corresponding disease. Specifically in psychiatric genetics, there have been numerous datasets from different platforms or sources such as association studies, including genome-wide association studies (GWAS), genome-wide linkage scans, microarray gene expression, and copy number variation, among others. Analyses of these datasets have led to many exciting discoveries, including disease susceptibility genes or loci, providing important insights into the underlying molecular mechanisms of the diseases. However, the results based on single domain data analysis are often inconsistent, with a very low replication rate in psychiatric disorders [1,2]. It has now been commonly accepted that psychiatric disorders, such as schizophrenia and major depressive disorder (MDD), have been caused by many genes, each of which has a weak or moderate risk to the disease [3,4]. Thus, a convergent analysis of multi-dimensional datasets to prioritize disease candidate genes is urgently needed. Such an approach may overcome the limitation of each single data type and provide a systematic view of the evidence at the genomic, transcriptomic, proteomic, metabolomic, and regulatory levels [5,6].

Recently, pathway and network-assisted analyses of genomic and transcriptomic datasets have been emerging as powerful approaches to analyze disease genes and their biological implications [7-11]. According to the observation of "guilt by association", genes with similar functions have been demonstrated to interact with each other more closely in the protein-protein interaction (PPI) networks than those functionally unrelated genes [12]. Similarly, we have seen accumulating evidence that complex diseases are caused by functional related genes (e.g., in pathways or protein complex) through their dynamic interaction and regulation rather than action by single gene alone. Taken together, a systematic analysis and comparison of disease genes in the PPI network would provide additional insights into the diseases that otherwise could not be identified by single gene or single marker analysis. It is important to note that, although network-based analysis has been widely applied in major complex diseases such as cancer, its application in psychiatric diseases has been limited so far.

MDD is a complex mental disorder with a lifetime prevalence of 9-19% [13-15] and moderate heritability (37-43%) [16]. Previous studies have suggested the involvement of polygenic and mutifactorial features in the pathology of MDD, as well as complex interactions among genes (G×G) and environmental factors (G×E) [17,18]. Recently, we have performed the first gene prioritization using multi-dimensional evidence-based datasets in MDD, including association, linkage, gene expression (both human and animal studies), regulatory pathway, and literature search (both human and animal studies) [19]. A list of depression candidate genes (which we named DEPgenes) with high reliability has been generated based on this strategy [19]. However, several characteristics remain unclear: the functional relationships among these DEPgenes, how they interact and regulate with each other, and how they act in the MDD. Such investigations are warranted for a deeper understanding of the molecular mechanisms of MDD but require comprehensive analysis at the systems biology level.

In this study, we first explored DEPgenes in the context of the PPI network for their topological characteristics and compared them with two representative complex diseases: schizophrenia and cancer. We performed the functional enrichment analyses using annotations from both Gene Ontology (GO) [20] and canonical pathways. More importantly, we examined crosstalk among the significantly enriched pathways by quantitatively measuring the shared protein components between each pair of pathways. Finally, we constructed a MDD-specific subnetwork using the DEPgenes and validated them using the association data from an independent GWAS dataset for MDD. Our work demonstrated a practical framework for complex disease candidate gene analysis at the functional level, which can be applied to other complex diseases.

## Materials and methods

### Depression candidate genes

We modified the scoring scheme in the gene prioritization system proposed by Kao *et al *[19] and reprioritized a list of 151 DEPgenes for MDD using the updated data information. Briefly, several lines of evidence-based datasets were collected for MDD, including association studies, linkage scans, gene expression (both human and animal studies), literature search (both human and animal studies), and biological regulatory pathways. A dataset-specific score was assigned for each gene in each data source, and all data types were combined by an optimized weighting matrix to indicate the priority of a gene's association with MDD. The final gene list was selected based on a set of previously implicated core genes for MDD and validated by the GWAS dataset. Detailed information of this gene prioritization procedure can be found in Kao *et al *[19]. Of note, the number of genes we used here is slightly different from that in Kao *et al *[19] due to the data and annotation updates, but the two lists were very similar.

### Other data sources and process

For the purpose of comparison, we collected schizophrenia candidate genes and cancer genes. Schizophrenia is a severe psychiatric disorder and has been suggested to share certain comorbidity with MDD clinically and genetically [21]. We included this disorder here to represent other psychiatric disorders for the purpose of comparison. We retrieved 160 schizophrenia candidate genes prioritized in our recent work using a similar multi-dimensional evidence-based strategy [22]. Cancer has been the most studied among all complex disease and is expected to have substantially different pathological features from MDD. Thus, it would be interesting to see how those genes act differently at the network and pathway levels. Cancer genes were downloaded from the Cancer Gene Census database [23] (CGC, July 2011).

The human PPI data was downloaded from the Protein Interaction Network Analysis (PINA) platform (downloaded in March 2010) [24], which collected and annotated data from six public PPI databases (MINT, IntAct, DIP, BioGRID, HPRD, and MIPS/MPact). Only proteins that could be successfully mapped to NCBI protein-coding genes were included in our analysis (see below). After removing self-interaction and duplicates, the final network included a total of 10,377 nodes and 50,109 interactions.

The GWAS dataset for major depression (dbGaP Study Accession: phs000020.v2.p1) was retrieved through our approved access to dbGaP [25]. We developed a pipeline for quality controls (QC) to the dataset. Detailed information can be found in our previous studies [19,26-28]. As a brief summary, there were 1,738 depression patients and 1,802 matched normal controls, and 424,861 markers after QC, covering a total of 16,758 genes. This dataset was used to evaluate the genes identified in this work.

To coordinate these heterozygous datasets in this study, we downloaded several key annotation files from the National Center for Biotechnology Information (NCBI) [29] for the ease of integration. These included the annotation files of *Homo_sapiens.gene_info.zip*, *gene_refseq_uniprotkb_collab.zip*, and *gene2refseq.zip *(as of November 24, 2010). DEPgenes, schizophrenia candidate genes, cancer genes, PPI data, and GWAS data were all mapped to human protein-coding genes from NCBI. Those genes that could not be mapped appropriately were discarded from the subsequent analysis.

### Network topological properties

In network analysis, there are several key topological indicators that have been defined to describe the behaviors or characteristics of the nodes in a network. The most representative ones are degree, betweenness, and shortest path. Degree is defined as the number of adjacent edges of a given node (protein) or the number of neighbor nodes interacting with it. Betweenness of a node is defined as the number of shortest paths going through the node; shortest path measures the nearest distance traveling from one node to another. We chose to examine the distribution of degree and betweenness of DEPgenes for exploration of their topological behaviors, and compared them with those of schizophrenia candidate genes [22] and cancer genes [30].

### Functional enrichment tests

To perform functional enrichment tests of the candidate genes, we used WebGestalt [31] for Gene Ontology (GO) term analysis and used the Ingenuity Pathway Analysis (IPA) system [32] for both canonical pathways and molecular networks. Although WebGestalt can perform enrichment tests for the Kyoto Encyclopedia of Genes and Genomes (KEGG) pathways [33], the IPA system provides a more comprehensive pathway resource based on manual collection and curation. The rich information returned by IPA is also suitable for pathway crosstalk analysis (see below), as it has more molecules and their connections included. Briefly, WebGestalt implements the hypergeometric test for the enrichment of GO terms in the candidate genes, followed by the correction of multiple testing using the Benjamini & Hochberg (BH) method [34]. The IPA system implements Fisher's exact test to determine whether a canonical pathway is enriched with genes of interest. Furthermore, the network analysis in the IPA system searches for significant molecular networks in a commercial knowledge base, including integrative information from literature, gene expression, and gene annotation.

### Pathway crosstalk

We performed pathway crosstalk analysis using the pathways that were significantly enriched with DEPgenes after multiple testing correction. Two pathways are considered to crosstalk if they share a proportion of DEPgenes. We introduced two measurements to computationally indicate the overlap of a pair of pathways: the Jaccard Coefficient $\text{JC}=\frac{\left|\text{A}\cap^{\text{B}}\right|}{\left|\text{A}\cup^{\text{B}}\right|}$ and the Overlap Coefficient $\text{OC}=\frac{\left|\text{A}\cap^{\text{B}}\right|}{\text{min}\left(\left|\text{A}\right|,\left|\text{B}\right|\right)}$, where *A *and *B *denote the number of candidate genes in the two pathways, respectively. To avoid non-specific inclusion of crosstalk, we further implemented the following rules: (1) only pathways with at least 5 DEPgenes were used; (2) only pathways with adjusted *P *values < 0.01 were used; and (3) two pathways in crosstalk were required to share at least 3 DEPgenes. These criteria were introduced to ensure that each of the pathways, as well as its crosstalk pair, have not only statistical significance but also a biologically meaningful number of genes, as some pathways may be too small. Finally, we found many significant pathways were identified by IPA; thus, they generated thousands of crosstalk events when all the pathway combinations were compared. In practice, we chose only those crosstalk events that had scores within the top 10% of the score distribution. Although these criteria were arbitrary, we found it worked efficiently to balance an appropriate number of pathways and crosstalk events.

### Construction of MDD-specific subnetwork

To construct a MDD-specific subnetwork, we applied the Steiner minimum tree algorithm that is implemented in our software framework *GenRev *[35] to the 151 DEPgenes. Solving the Steiner minimum tree algorithm was proposed by Klein and Ravi [36], which can be used for constructing a connected subnetwork given a list of query nodes. In our case, the query nodes are those encoded by DEPgenes, and the whole network is the human interactome extracted from the PINA database (see above). This algorithm aims to connect a maximum proportion of the query nodes. To accomplish this, additional nodes in the network, but not in the query list, would be recruited in order to make the target subnetwork interconnected, while the algorithm is optimized towards a minimum list of the additional nodes. *GenRev *is a recently developed software tool which implements the Steiner minimum tree algorithm, as well as two other popular algorithms for subnetwork construction. It has been successfully applied in our previous work [6,22,37]. In the work discussed here, we used it for DEPgenes to construct MDD-specific subnetwork.

## Results

### Network topological properties of depression genes

**Figure 1:**
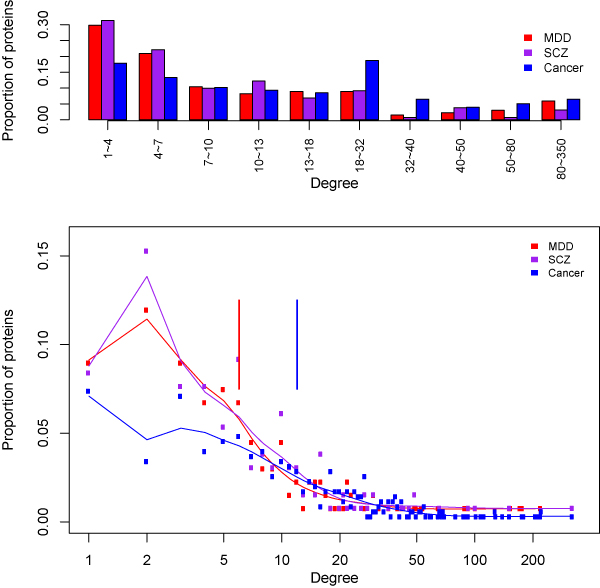
**Comparison of degree distribution of major depressive disorder (MDD), schizophrenia (SCZ), and cancer genes**. The disease genes were grouped by their degree into degree bins. Here, degree was measure by the number of interactors for each disease gene in the human interactome. The top panel shows the histogram degree distribution, and the bottom panel shows the curve degree distribution. In the bottom panel, each vertical line represents the median value of the degrees in each disease category. Note that MDD and SCZ candidate genes had the same median value of degrees so that their vertical lines could not be distinguished.

We collected 151 major depressive disorder candidate genes (DEPgenes). Among them, 134 had protein interaction annotations in the human interactome. Figure 1 shows the degree distribution. The average degree of these proteins was 18.55, and their median degree value was 6. As a comparison, the average degree was 14.75 (median value 6) for the schizophrenia candidate genes (131 of the 160 genes mapped onto the human interactome) and 25.53 (median value 12) for the cancer genes (353 of the 459 genes mapped onto the interactome). Overall, although DEPgenes on average had a higher degree value than schizophrenia genes, their degree distribution is similar to that of schizophrenia genes, and statistical tests indicated no significant difference (Wilcoxon test, *P *= 0.53). However, we observed different degree distributions between DEPgenes and cancer genes, and statistical tests indicated that DEPgenes had significantly lower degrees than cancer genes (*P *= 1.93 × 10^-5^). Specifically, cancer genes were found more frequently in the degree bins 18-32 and 32-40 (Figure 1).

For the measurement of betweenness, the average value was 5.02 × 10^4 ^for DEPgenes, 4.01 × 10^4 ^for the schizophrenia genes, and 5.61 × 10^4 ^for cancer genes, while their median values were 5.12 × 10^3^, 3.54 × 10^3^, and 1.02 × 10^4^, respectively. Similar to the measurement of degree, there was no significant difference in the betweenness values between the MDD and schizophrenia candidate genes (*P *= 0.21), but cancer genes had significantly larger betweenness values than DEPgenes (*P *= 0.03). These results indicated that the candidate genes for the two major psychiatric disorders, MDD and schizophrenia, shared similar topological features in the human interactome, while both had substantially different features when compared to cancer genes.

### Gene Ontology enrichment analysis by WebGestalt

To explore whether DEPgenes share specific functional features, we performed GO enrichment analysis using WebGestalt (version 2.0). We found that many neurodevelopment related functions and biological processes were significantly enriched in DEPgenes, regardless of GO terms categories (BP: biological process; MF: molecular function; and CC: cellular component) (Table 1). The most significant terms in each of these three GO categories are: synaptic transmission in biological process (*P*_BH _= 1.18 × 10^-34^), G-protein coupled amine receptor activity in molecular function (*P*_BH _= 7.18 × 10^-19^), and neuron projection in cellular component (*P*_BH _= 3.91 × 10^-20^). Other enriched GO terms of interest include transmission of nerve impulse, neurological process, cell communication, dopamine binding, extracellular ligand-gated ion channel activity, ligand-gated channel activity, axon, and dendrite.

**Table 1:** Gene Ontology (GO) terms enriched with module genes (GO level ≥ 4)

GO terms	Observed*	*P*	*P*_BH_^$^
*Biological process*			
GO:0007268: synaptic transmission	45	7.18 × 10^-38^	1.18 × 10^-34^
GO:0007267: cell-cell signaling	56	8.78 × 10^-37^	7.21 × 10^-34^
GO:0019226: transmission of nerve impulse	46	2.84 × 10^-36^	1.55 × 10^-33^
GO:0044057: regulation of system process	36	1.23 × 10^-29^	2.89 × 10^-27^
GO:0051239: regulation of multicellular organismal process	55	1.07 × 10^-29^	2.89 × 10^-27^
GO:0050877: neurological system process	59	4.72 × 10^-26^	9.69 × 10^-24^
GO:0007154: cell communication	103	6.14 × 10^-26^	1.12 × 10^-23^
*Molecular function*			
GO:0008227: G-protein coupled amine receptor activity	15	5.13 × 10^-21^	7.18 × 10^-19^
GO:0035240: dopamine binding	7	2.25 × 10^-13^	1.58 × 10^-11^
GO:0005230: extracellular ligand-gated ion channel activity	12	4.17 × 10^-12^	2.34 × 10^-10^
GO:0004888: transmembrane receptor activity	40	6.55 × 10^-12^	2.78 × 10^-10^
GO:0005102: receptor binding	31	9.87 × 10^-11^	3.07 × 10^-9^
GO:0022834: ligand-gated channel activity	13	2.46 × 10^-10^	5.74 × 10^-9^
*Cellular component*			
GO:0043005: neuron projection	30	2.43 × 10^-22^	3.91 × 10^-20^
GO:0044459: plasma membrane part	61	3.12 × 10^-20^	2.51 × 10^-18^
GO:0000267: cell fraction	45	1.45 × 10^-19^	7.78 × 10^-18^
GO:0005887: integral to plasma membrane	47	5.84 × 10^-19^	2.35 × 10^-17^
GO:0031226: intrinsic to plasma membrane	47	1.27 × 10^-18^	4.09 × 10^-17^
GO:0042995: cell projection	35	5.50 × 10^-18^	1.48 × 10^-16^
GO:0005886: plasma membrane	80	1.64 × 10^-17^	3.77 × 10^-16^
GO:0030424: axon	18	1.15 × 10^-15^	2.31 × 10^-14^
GO:0030425: dendrite	17	2.76 × 10^-14^	4.94 × 10^-13^
GO:0005626: insoluble fraction	32	7.11 × 10^-13^	1.14 × 10^-11^

*Number of the observed DEPgenes in the category.

^$^*P *values were adjusted by Benjamini & Hochberg (BH) method [34].

### Pathway enrichment by Ingenuity Pathway Analysis

We then examined whether DEPgenes are enriched in canonical pathways by performing Fisher's exact test in the IPA system. Table 2 shows the 12 most significantly enriched pathways. Remarkably, most of them are related to the neurotransmission system, supporting the neuropathology hypothesis of MDD (Table 2). Among them, we highlighted serotonin receptor signaling, dopamine receptor signaling, PXR/RXR activation, neuropathic pain signaling on dorsal horn neurons, CREB signaling in neurons and tryptophan metabolism. This result is consistent with prior knowledge of MDD [38,39], providing further evidence of the neuro-related processes in this disorder.

**Table 2:** Canonical pathways enriched with module genes by Ingenuity Pathway Analysis (IPA) (*P*_BH _< 10^-6^)

Ingenuity canonical pathways	Observed*	*P*_BH_^$^
cAMP-mediated signaling	23	6.31 × 10^-16^
G-protein coupled receptor signaling	31	5.01 × 10^-15^
Serotonin receptor signaling	12	6.31 × 10^-15^
Corticotropin releasing hormone signaling	14	7.94 × 10^-11^
Dopamine receptor signaling	11	3.80 × 10^-9^
Glucocorticoid receptor signaling	17	1.35 × 10^-8^
PXR/RXR activation	10	2.29 × 10^-8^
Amyotrophic lateral sclerosis signaling	11	5.89 × 10^-8^
Neuropathic pain signaling on dorsal horn neurons	11	5.89 × 10^-8^
Relaxin signaling	12	1.02 × 10^-7^
CREB signaling in neurons	13	1.62 × 10^-7^
Tryptophan metabolism	11	6.61 × 10^-7^

*Number of the observed DEPgenes in the category.

^$^*P *values were adjusted by Benjamini & Hochberg (BH) method [34].

### Crosstalk among significantly enriched pathways

Since many genes and pathways might be involved in MDD, to more deeply understand how these pathways are related, we performed a pathway crosstalk analysis. We first selected the significantly enriched pathways from the IPA results. Specifically, we selected those pathways having *P*_BH _< 0.01 and ≥ 5 DEPgenes. There were 71 pathways that met these criteria. Among them, 69 pathways shared at least 3 genes with other pathways. A total of 571 edges (links) connected between any two of these pathways, and these edges were ranked according to the average scores of the Jaccard Coefficient and the Overlap Coefficient (see the Materials and methods section). We selected the top 10% edges, which resulted in 57 pairs of pathway crosstalk, and constructed the pathway crosstalk network for MDD. This pathway crosstalk was the first of its kind in MDD.

**Figure 2:**
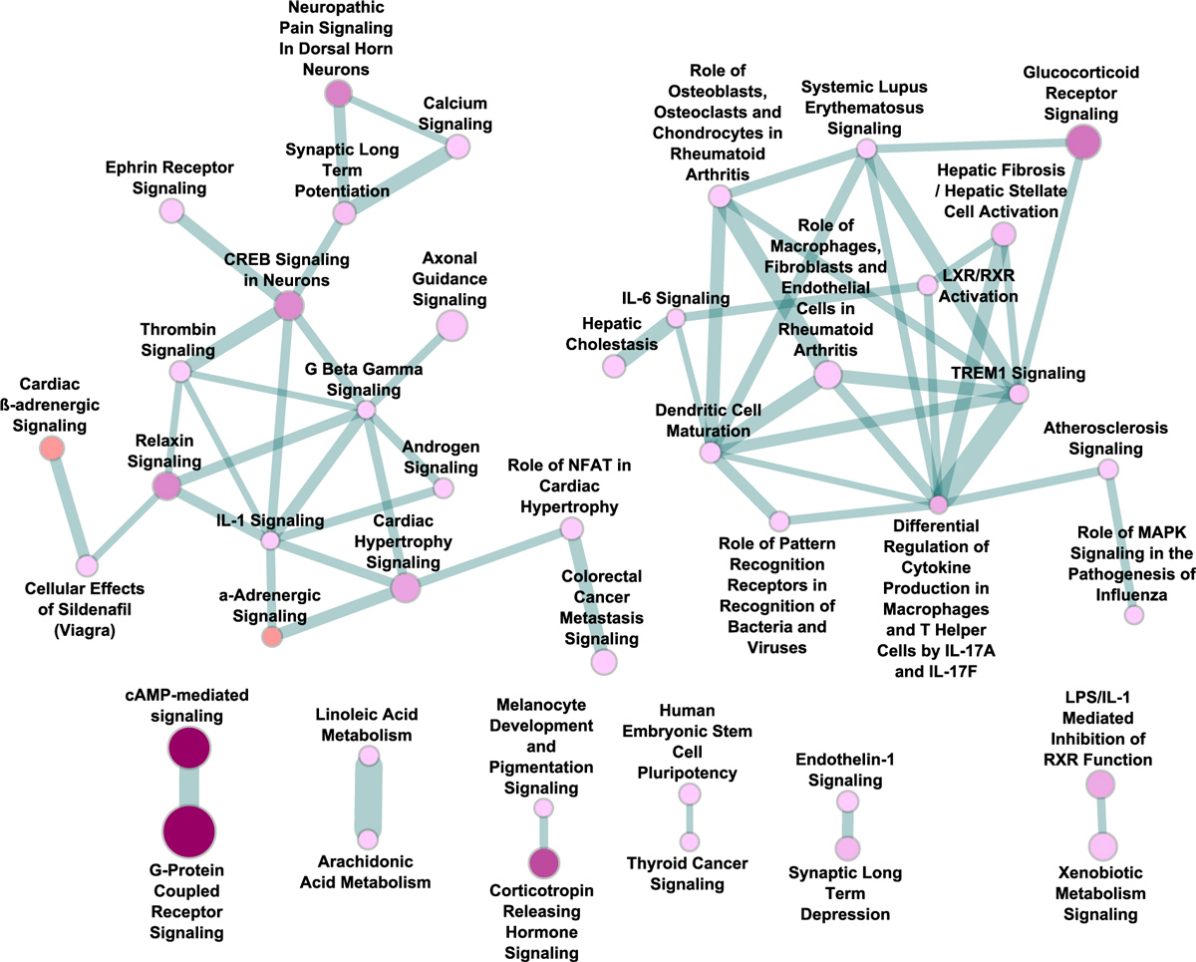
**Pathway crosstalk and functional map of DEPgenes (major depressive disorder genes)**. In this figure, each node represents a significant pathway, and each edge represents a pathway crosstalk, i.e., a significant overlap of the component genes between two linked pathways. The color of each node is approximately proportional to the adjusted *P *(*P*_BH_) value of the corresponding pathway in the pathway enrichment analysis by Ingenuity Pathway Analysis (IPA). Darker color indicates lower *P*_BH _value. The size of each node is approximately proportional to the number of DEPgenes found in the corresponding pathway. The width of each edge is approximately proportional to the overlap score of the related pathways (see Materials and methods).

Graphical presentation of the selected pathway crosstalk revealed two self-clustered modules, as well as small but strongly-linked pathway pairs. As shown in Figure 2, the two large modules are dominated by neuro-related signal transduction and immune related pathways, respectively. The neuro-related signal transduction module consists of the calcium signaling pathway, synaptic long term potentiation, CREB signaling in neurons, axonal guidance signaling, and others. These pathways have been well studied and have been indicated in many psychiatric disorders that share comorbidity with MDD [5,40,41].

The second large pathway crosstalk module mainly consisted of immune-related pathways, such as IL-6 signaling and LXR/RXR activation (Figure 2). This strongly supports recent discoveries of immunity and inflammation related processes in psychiatric disorders [42,43], including MDD [44]. Many genes that drove the crosstalk in the figure were also found to function in both neuro- and immune-related processes like *APOE *[45], *TNF *[46], and *IL6 *[47].

### Molecular subnetwork

**Figure 3:**
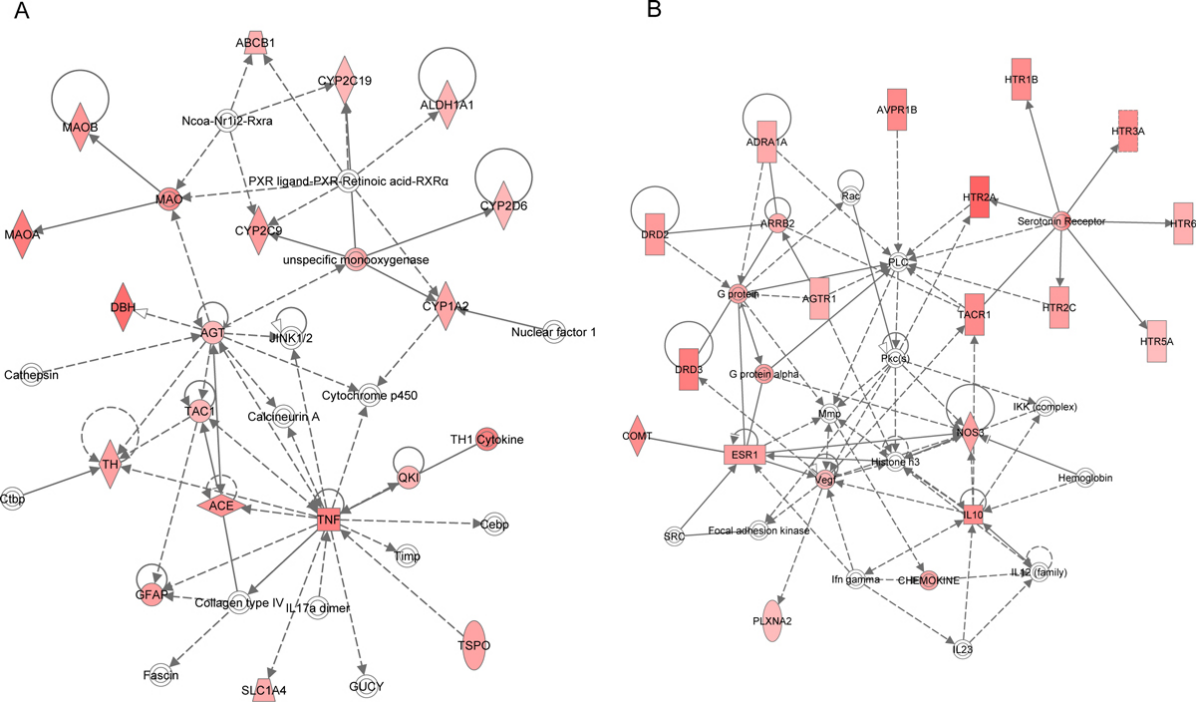
**The top two molecular networks identified by Ingenuity Pathway Analysis (IPA)**. (A) The most significant molecular network by IPA pathway enrichment analysis. (B) The second most significant molecular network. Color of each node indicates the score of each DEPgene calculated by multiple lines of genetic evidence, as described in Kao *et al *[19].

A total of 8 significant molecular networks were identified by Fisher's exact test in the IPA system with additional criteria specifying that a pathway's score was at least 10 and each pathway had at least 10 DEPgenes. Here, score was transformed from -log*P*, where *P *is calculated by the Fisher's exact test. Figure 3 showed the two most significant networks, in which DEPgenes were highlighted in red. In the first network (Figure 3A), we observed 18 DEPgenes, and the top functions of this network included energy production, drug metabolism, and small molecule biochemistry. The second network, which consisted of 18 DEPgenes too, was enriched with the functions of genetic disorder, neurological disease, and psychological disorders. On the molecular level, we observed a group of serotonin receptors and G-proteins (Figure 3), further supporting the involvement of neurological signaling in major depressive disorder.

### MDD-specific subnetwork

**Figure 4:**
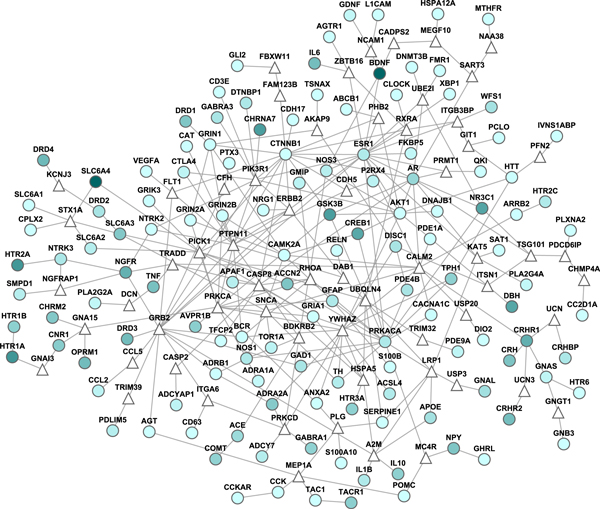
**Major depressive disorder (MDD) specific protein-protein interaction subnetwork**. Round nodes are DEPgenes (MDD candidate genes) and triangular nodes are additional genes recruited by subnetwork construction. The darkness of node color is approximately proportional to the integrative evidence score of each DEPgene, as described in Kao *et al *[19].

**Figure 5:**
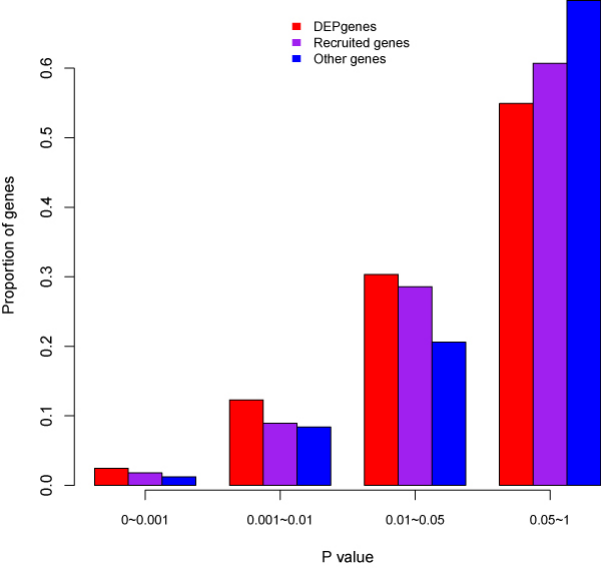
**Comparison of the distribution of GWAS *P *values in three gene sets: DEPgenes in the MDD-specific subnetwork, non-DEPgenes recruited in the MDD-specific subnetwork, and other genes examined in the GWAS dataset**. X-axis is the gene-wise *P *value grouped into four bins, and Y-axis is the proportion of genes in the corresponding *P *value bin.

Among the 151 DEPgenes, 134 were found to have PPI annotations in the human interactome. Using our recently developed subnetwork extraction tool *GenRev*, we successfully constructed a MDD-specific subnetwork. The subnetwork contained 130 DEPgenes and 62 additional genes that were recruited via the subnetwork construction algorithm (Steiner minimum tree algorithm [36]) (Figure 4). To evaluate the genes identified in the subnetwork, we compared their *P *values in a GWAS dataset for MDD (see the Materials and methods section). Among the 16,758 genes in the MDD GWAS dataset, we had 122 DEPgenes in the subnetwork, 56 non-DEPgenes in the subnetwork (we named them subnetwork's recruited genes), and remaining 16,580 genes outside of the subnetwork. For each gene, we assigned a gene-wise *P *value based on the SNP that had the smallest *P *value among all the SNPs mapped to the gene region [4,26]. When we separated gene-wise *P *values into four bins (<0.001, 0.001-0.01, 0.01-0.05, and ≥0.05), we found both the DEPgenes and the newly recruited genes in the subnetwork were more frequent in the small *P *value bins (<0.001, 0.001-0.01, 0.01-0.05) than other genes (Figure 5). Furthermore, DEPgenes tended to have smaller gene-wise *P *values than the newly recruited genes, supporting that subnetwork analysis could identify potential disease genes that would otherwise unlikely be detected by traditional singe gene or single marker association studies. When using cutoff value 0.05 to separate the genes into three gene sets (i.e., nominally significant genes were defined as those with gene-wise *P *value < 0.05), we found that the DEPgenes in the subnetwork had a significantly larger proportion of nominally significant genes in the GWAS dataset (Fisher's exact test, *P *= 4.13 × 10^-4^) compared to the remaining genes. The recruited genes in the subnetwork were found to have a similar trend of larger proportion of nominally significant genes than remaining genes, but this difference was not significant (*P *= 0.10). Of note, when comparing the genes in the MDD-specific subnetwork (122+56 = 178 genes) with those outside of the network (16,580 genes), the subnetwork genes had significantly more nominally significant genes (*P *= 1.81 × 10^-4^).

## Discussion

Although there have been numerous reports of susceptibility genes or loci to psychiatric disorders such as major depressive disorder and schizophrenia, no disease causal genes have been confirmed [48-50]. One important task now is to reduce the data noise and prioritize the candidate genes from multiple dimensional genetic and genomic datasets that have been made available during the last decade and then explore their functional relationships for further validation. To our knowledge, this is the first systematic network and pathway analysis for MDD using candidate genes prioritized from comprehensive evidence-based data sources. By overlaying the MDD candidate genes in the context of the human interactome, we examined the topological characteristics of these genes by comparing them with those of schizophrenia and cancer candidate genes. We further performed pathway enrichment analysis to better understand the biological implications of these genes in the context of the regulatory system. Building on our observation of the large number of pathways enriched with DEPgenes, we developed novel approaches to measure pathway crosstalk so that complex gene action and regulation could be explored, thus providing us new insights into the interpretation of the underlying molecular mechanisms in MDD.

Our network topological analysis revealed that DEPgenes showed similar topological characteristics to schizophrenia, supporting previous reports that depression and schizophrenia might share comorbidity both clinically and genetically [21]. For example, clinical symptoms such as psychosis and neuro-cognitive impairments have been observed in both depression and schizophrenia patients [21], and shared genetic variance has been reported between major depression and schizophrenia [51,52]. Although similar network topological features are expected by many investigators, our study was the first to confirm, and provided further evidence, that the topological features of depression genes are different from cancer genes. It is worth noting that, although depression and schizophrenia genes had similar degree distributions (Figure 1), depression genes had moderately stronger connectivity and betweenness than schizophrenia genes.

Of significance, our pathway crosstalk analysis revealed two large clustered modules, both of which had important implications to MDD (Figure 2). The first cluster included 17 pathways, and it was dominated by neuro-signaling pathways. Among these pathways, neuropathic pain signaling in dorsal horn neurons (*P*_BH _= 5.89 × 10^-8^), CREB signaling in neurons (*P*_BH _= 1.62 × 10^-7^), synaptic long term potentiation (*P*_BH _= 6.17 × 10^-5^), and axonal guidance signaling (*P*_BH _= 1.55 × 10^-4^) are involved in neuron/brain tissues and have been reported to be involved in MDD [53,54]. Our further examination of the genes contributing to the crosstalk revealed that the most frequently shared genes in this cluster were *PRKACA *(functioning in n = 15 pathways in this cluster), *GNAS *(n = 14), *GNB3 *(n = 13), *ADCY7 *(n = 10), *GNAL *(n = 9), *AKT1 *(n = 9), *CREB1 *(n = 8), *CAMK2A *(n = 6), *GRIN2B *(n = 5), *GRIN2A *(n = 5), and *GRIN1 *(n = 5), among others.

The second cluster is primarily related to immunity and inflammation, including the IL-6 signaling pathway (*P*_BH _= 6.17 × 10^-3^), differential regulation of cytokine production in macrophages and T helper cells by IL-17A and IL-17F (*P*_BH _= 8.13 × 10^-6^), and LXR/RXR activation (*P*_BH _= 4.57 × 10^-4^). For example, the LXR/RXR pathway may play a role in the prevention of programmed cell death and a role in immune responses to inhibit inflammatory gene expression [55]. The most frequently shared genes in this cluster included *TNF *(functioning in n = 14 pathways), *IL6 *(n = 13), *IL1B *(n = 13), *IL10 *(n = 9), *CCL2 *(n = 8), *NGFR *(n = 7), and *AKT1 *(n = 7), among others. These genes further support the observation that immune- and inflammation-related functions are involved in this cluster. During recent years, evidence of immune and inflammation systems in psychiatric disorders has accumulated quickly [3,4,56].

In addition to the two major clusters, there are other crosstalk pairs that are noteworthy. The most interesting one is the pathway pair of cAMP-mediated signaling and G-protein coupled receptor signaling. The evidence linking these two pathways is strong, as its edge had a score 0.87. Moreover, these two pathways had the most significant enrichment test *P *values (6.31 × 10^-16 ^and 5.01 × 10^-15^, respectively) in the IPA canonical pathway analysis (Table 2). The interaction between these two pathways involved 23 DEPgenes, including several serotonin receptor genes like *HTR1A*, *HTR1B*, and *HTR5A*. The cAMP-mediated signaling and G-protein coupled receptor signaling pathways have long been studied for their roles in the nervous system. Of note, there were several crosstalk links between one of these two pathways and other pathways that were enriched with the DEPgenes. Those pathway crosstalk connections were not shown in Figure 2 because they did not meet our stringent criteria for pathway inclusion (at least 3 DEPgenes shared between the pair of pathways or not within the top 10% crosstalk score, see the Materials and methods section). One example is the link between the cAMP-mediated signaling pathway and the serotonin receptor signaling, both of which were significantly enriched with DEPgenes, but their crosstalk score fell outside of the top 10% in the score distribution.

Our aim of the depression-specific subnetwork construction was to explore functional interactions of DEPgenes in a local protein-protein interaction environment. Our follow-up evaluation of the disease association of both DEPgenes and the additionally recruited genes using a major GWAS dataset for depression found that these genes tended to have small *P*-values (i.e., at the nominal significance level). Since the GWAS data we used here was an independent dataset, and GWAS was designed to be hypothesis free in genome-wide association studies, our survey of MDD-specific subnetwork genes demonstrated that this approach is efficient to find a set of genes that are both functionally interactive and enriched with the association signals of the corresponding disease. Therefore, this approach is not only promising to find novel disease candidate genes for future validation but also useful to study the disease at the systems biology level.

This work has a few limitations. First, our DEPgenes and the follow up pathway/network analyses were conducted based on computational strategies. Although informative, this approach generally requires extensive experimental validation. Thus, although we validated subnetwork genes at the genome-wide level using the GWAS dataset, further validation of specific novel genes using more samples is urgently needed. Second, the pathway crosstalk analysis was based on the scores measured by Jaccard Coefficient (*JC*) and Overlap Coefficient (*OC*). In this study, we selected the pathway pairs empirically, that is, those ranked in the top 10%. *P *values from a statistic test would be better applied to select significant crosstalk. We did not apply this method because the Ingenuity Pathway Analysis system is a commercial software tool, and the information needed to conduct such a statistic test is not publically available. Accordingly, we could only use the limited information for pathway crosstalk analysis. Third, the MDD-specific subnetwork was built on available human interactome data. Although the number and quality of protein interactions has recently improved greatly, the human interactome is still incomplete with many false positives [24]. Additionally, subnetwork extraction relies on specific algorithms and corresponding parameters. Several algorithms exist for subnetwork extraction. In this study, we applied the Steiner minimum tree algorithm, which can effectively reduce unrelated nodes (genes) to be included, but it may also miss some important functional links. Our analysis, along with our recent application of this algorithm in other complex diseases (schizophrenia [5,57], hepatocellular carcinoma [37], and epilepsy [6]), has demonstrated this strategy is practical and could provide valuable information of the interactions among DEPgenes.

## Conclusions

We developed a systems biology framework for advanced and functional analyses of major depressive disorder candidate genes. The network topological analysis revealed similar network characteristics between depression and schizophrenia, but network characteristics of both depression and schizophrenia differed from cancer, consistent with previous clinical and genetic studies. However, the depression genes interacted moderately stronger than schizophrenia genes in the context of the protein-protein interaction network. Our pathway enrichment tests followed by pathway crosstalk analysis revealed that neurotransmission and immune systems might play key roles in the etiology of depression, assuming that our evidence-based DEPgenes were representative of depression. Notably, we found two major functional clusters in the pathway crosstalk network. We further constructed a depression-specific subnetwork, in which additional candidate genes were identified with enriched association signals using the depression GWAS dataset. These findings present a wealth of information for future validation. The framework we presented in this work can be applied to many other complex diseases, such as addiction and bipolar disorder.

## Competing interests

The authors declare that they have no competing interests.

## Authors' contributions

PJ, CK, PK and ZZ conceived and designed the experiments. PJ carried out the data analysis. PJ, CK, PK and ZZ drafted the manuscript. All authors read and approved the final manuscript.
